# Modeling miRNA-mRNA interactions that cause phenotypic abnormality in breast cancer patients

**DOI:** 10.1371/journal.pone.0182666

**Published:** 2017-08-09

**Authors:** Sanghoon Lee, Xia Jiang

**Affiliations:** Department of Biomedical Informatics, University of Pittsburgh, Pittsburgh, Pennsylvania, United States of America; University of São Paulo, BRAZIL

## Abstract

**Background:**

The dysregulation of microRNAs (miRNAs) alters expression level of pro-oncogenic or tumor suppressive mRNAs in breast cancer, and in the long run, causes multiple biological abnormalities. Identification of such interactions of miRNA-mRNA requires integrative analysis of miRNA-mRNA expression profile data. However, current approaches have limitations to consider the regulatory relationship between miRNAs and mRNAs and to implicate the relationship with phenotypic abnormality and cancer pathogenesis.

**Methodology/Findings:**

We modeled causal relationships between genomic expression and clinical data using a Bayesian Network (BN), with the goal of discovering miRNA-mRNA interactions that are associated with cancer pathogenesis. The Multiple Beam Search (MBS) algorithm learned interactions from data and discovered that hsa-miR-21, hsa-miR-10b, hsa-miR-448, and hsa-miR-96 interact with oncogenes, such as, CCND2, ESR1, MET, NOTCH1, TGFBR2 and TGFB1 that promote tumor metastasis, invasion, and cell proliferation. We also calculated Bayesian network posterior probability (BNPP) for the models discovered by the MBS algorithm to validate true models with high likelihood.

**Conclusion/Significance:**

The MBS algorithm successfully learned miRNA and mRNA expression profile data using a BN, and identified miRNA-mRNA interactions that probabilistically affect breast cancer pathogenesis. The MBS algorithm is a potentially useful tool for identifying interacting gene pairs implicated by the deregulation of expression.

## Introduction

One of the important roles of microRNAs (miRNAs) is gene silencing that is carried out by post-transcriptional repression of target mRNA expression [1]. The aberrance of mRNA expression level disturbs the modulation of diverse biological processes and pathogenesis in human diseases. In fact, an increasing amount of evidence demonstrated that miRNA expression profiles are dysregulated in human breast cancer and they mediate phenotypic characteristics such as tumor cell growth, migration, metastasis, and invasion [2–5]. The extensive use of miRNA microarray and RNA-seq data has supported that miRNAs can be potential biomarkers to predict treatment response, tumor progression, and survival time [6–8]. Although a role of miRNA expression in cancer biology has not been fully established, the miRNA expression profile is used to classify tumor subtypes according to clinico-pathological features, such as expression level of hormone receptors, tumor grade, and molecular subtypes in breast cancer [9, 10]. It is important to identify the effects of miRNAs on the expression level of target genes within the pathogenesis of human cancer.

### The effect of miRNA on the mRNA destabilization and breast cancer pathogenesis

At the time of initial research in this area, it was unknown how miRNA exerts its effect on translation control. It was thought that inhibition of mRNA translation and down-regulation of mRNA by destabilization are not related to each other [11–13]. However, biochemical and genomic studies using microarray and RNA-seq demonstrated that direct repression of mRNA translation and destabilization of mRNA are synchronous in the initiation phase of miRNA action [14, 15], and interestingly proteins that were repressed in translation by miRNAs also had remarkable down-regulation in their corresponding mRNAs [16]. Further studies concluded that reduced protein translation is mainly through the destabilization of target mRNAs, and direct inhibition to translation by miRNA has rather moderate impact [17, 18]. This prevailing mechanism of miRNA action explained why miRNAs cause extensive transcriptional changes in multicellular organisms through the interaction with mRNAs, and implies that a miRNA-mRNA interaction does not mean a simple base-paring complementarity between two mRNAs. Rather, the interaction means broad transcriptional changes and the regulation of protein translation. Measurement of the substantial transcriptional changes caused by miRNA modulation is necessary to discover and validate miRNA-mRNA interactions. Therefore, genomic profiling using microarray or RNA-seq should be a good approach to measuring the expression changes in mRNA and finding the association between miRNAs and mRNAs.

The effect of the deregulation of miRNA expression on the cancer pathogenesis is presumed to be implicated with the alterations of target mRNA expression level [19]. An increasing amount of evidence demonstrated that the alterations of miRNA and target mRNA expression are correlated with human cancers. Volinia et al. [20] identified miRNA signature overexpressed in human solid tumor and got the list of putative target mRNAs for the identified miRNA signature based on the information of TargetScan database. TargetScan is a database that users type miRNA names and search for predicted biological targets of the miRNAs [21]. Among the putative target mRNAs, they could specify known tumor genes, such as RB1 (Retinoblastoma 1, tumor suppressor) and TGFBR2 (transforming growth factor, oncogene). On the other hand, Zhu et al. [22] observed that tumor invasion and metastasis were significantly reduced when hsa-mir-21 was suppressed in human breast cancer cell line. hsa-miR-21 is known to act as an oncogenic miRNA that is overexpressed in human cancers and induce tumor growth [10, 23]. The tumor progression reduced by hsa-miR-21 down-regulation is mediated by the hsa-miR-21 target genes; the target genes are metastasis-related tumor suppressor genes such as TPM1, Programmed Cell Death 4 (PDCD4) and SERPINB5. It is important to understand how the functions of miRNAs and their effects on the expression level alteration of target genes are integrated within the pathogenesis of human cancer.

### The mechanisms of miRNA-mRNA interaction

The basic biological function of miRNAs is that they interact with target complementary mRNAs to interfere with the expression level of mRNAs, which can be encoding proteins or factors that control developmental and physiological process in plants and animals [24]. The predominant effects of miRNAs are to achieve post-transcriptional regulation such as repressing protein synthesis at initiation or during elongation, mRNA destabilization, and cleavage of mRNA transcripts [25]. Understanding the effect of miRNAs on translation is important because protein synthesis is one of the most important biological processes for phenotype. Of the many mechanisms to explain the predominant effects, two main ones are direct inhibition of protein translation [26, 27], and indirect destabilization of mRNAs, where miRNAs curtail the poly-A tails of mRNAs to degrade them and reduce protein translation [28, 29]. It is considered that translation repression primarily occurs given imperfect complementarity between the miRNAs and target mRNAs in ‘seed’ region (nucleotides 2~7 in 5′ end of miRNAs). The argonaute protein, Ago2, leads to direct mRNA degradation in the RNA induced silencing complex (RISC) [30]. It has been unclear how much miRNAs exert on the control of translational inhibition directly compared to mRNA destabilization. Selbach et al. [11] demonstrated that miRNAs also directly repress the translation of hundreds of genes by using a proteomic approach to measure the changes in synthesis of proteins on a genome-wide scale, and compared the result with quantified mRNA levels in microarray experiment. One thing clear is that miRNAs can tune protein synthesis from thousands of mRNAs through translation inhibition directly or RNA degradation indirectly.

The comprehensive association of miRNAs with human cancers occurs by regulating tumor oncogenes, suppressor genes, or transcription factors in a signaling pathway [31]. Due to the regulatory role of miRNAs on tumor-associated genes, incorporating the aberrant miRNA expressions with the consequential alteration of expression profile in target mRNAs is a good approach for discovering their interactions that have a combined effect on a cancer pathogenesis [19]. Several studies have proposed to identify miRNA-mRNA interactions from expression data using computational approaches and models, such as Bayesian inference from sequencing and expression data [32], and probabilistic graphical model [33, 34]. However, most of existing methods did not consider the relationship between the group of miRNAs and the group of target mRNAs in genomic regulatory networks. In addition, incorporation of miRNA and mRNA expression profile data with clinical data has been one of main challenges to find miRNA-mRNA interactions specifically associated with human cancer.

Identifying the interactions between miRNAs and mRNAs in malignant tumors holds promise towards developing cancer diagnostic and prognostic biomarkers. The Bayesian Network is an effective architecture for representing statistical dependencies and modeling causal relationships among genes and clinical features. In this study, we modeled the interaction of miRNA and mRNA expression to affect breast cancer pathogenesis using a Bayesian Network. We then identified interactions that have causal effect on cancer pathogenesis by applying an efficient search algorithm to datasets concerning breast cancer. Next, we briefly review Bayesian Networks.

### Bayesian networks

**Fig 1:**
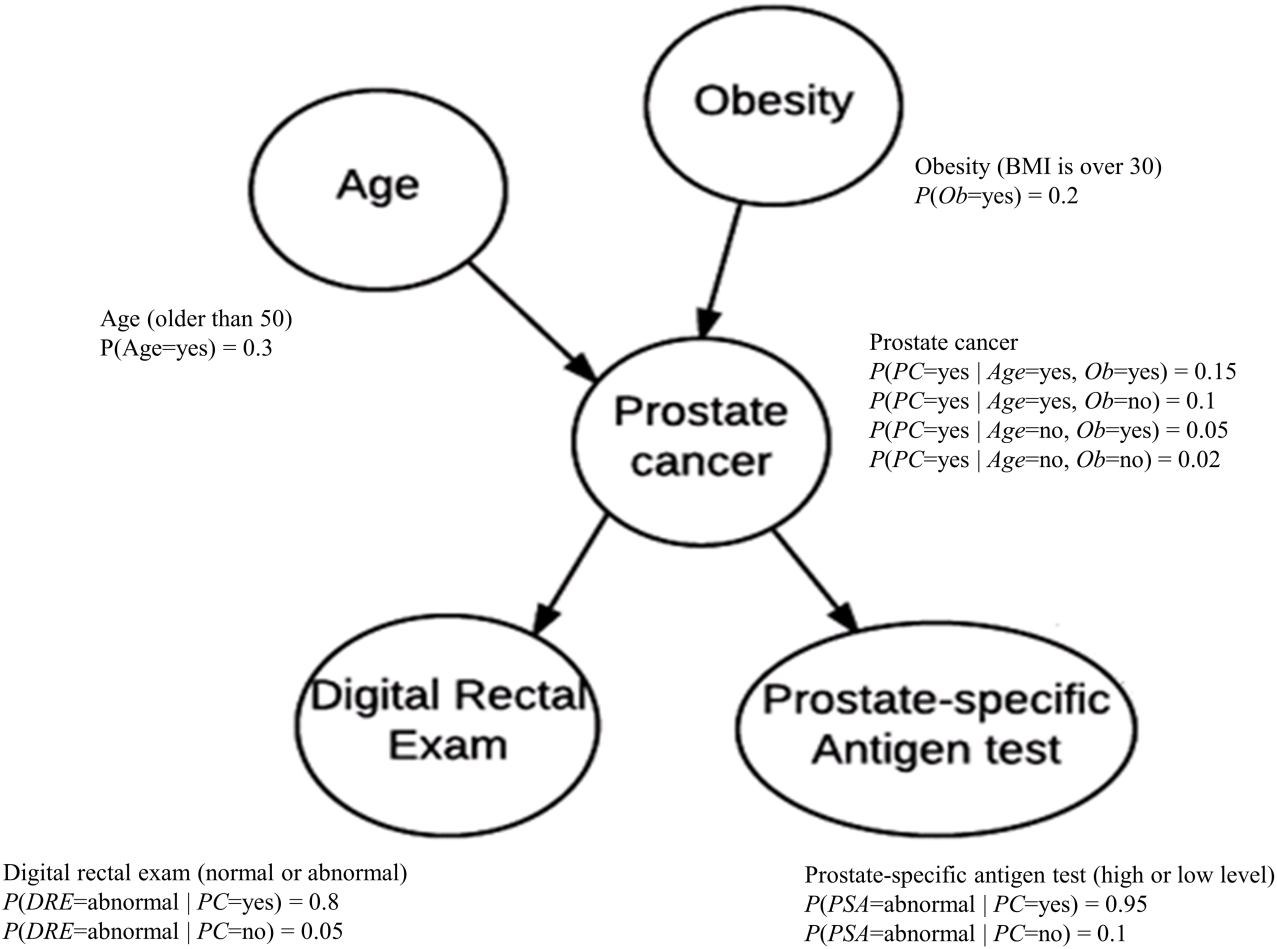
A BN representing relationships among variables related to prostate cancer.

A Bayesian Network (BN) is a leading architecture for representing uncertainty in computational intelligence and has been applied to many domains including biomedical informatics [35–38]. A BN consists of a directed acyclic graph (DAG) *G* = (*V*, *E*), whose node set *V* contains random variables and whose edge set *E* represents relationships among the variables, and a conditional probability distribution of each node *X* ∈ *V* given each combination of values of its parents. Each node *V* in a BN is conditionally independent of all its non-descendants given its parents. Often the DAG in a BN is a causal DAG [39]. Fig 1 shows an example of BN modeling relationships among variables related to prostate cancer.

The task of learning a BN from data concerns learning both the parameters in a BN and the structure (called a DAG model). In a score-based structure learning approach, we assign a score to a DAG based on how well the DAG fits the data. Cooper and Herskovits [40] introduced the Bayesian Dirichlet equivalent uniform (BDeu) score, which calculates the probability of the data given the model *G*. In the BDeu score calculation, the users can specify priors for the conditional probability distributions using a single hyperparameter α, called the prior equivalent sample size for conditional probability distributions. That score formula is as follows:

$$score_{\alpha }(G\;:\;Data)=P(Data|G)=\overset{n}{\underset{i=1}{\Pi }}\overset{q_{i}}{\underset{j=1}{\Pi }}\frac{\Gamma (\alpha /q_{i})}{\Gamma (\alpha /q_{i}+\Sigma_{k=1}^{r_{i}}s_{ijk})}\overset{r_{i}}{\underset{k=1}{\Pi }}\frac{\Gamma (\alpha /r_{i}q_{i}+s_{ijk})}{\Gamma (\alpha /r_{i}q_{i})}$$ (1)

Where *n* is the number of nodes in G, *r*_*i*_ is the number of states of node *X*_*i*_, *q*_*i*_ is the number of different instantiations of the parents of *X*_*i*_, and *s*_*ijk*_ is the number of times in the data that *X*_*i*_ took its *k* th value when the parents of *X*_*i*_ had their *j* th instantiation.

### The Multiple Beam Search (MBS) algorithm

Jiang et al. [41] developed the Multiple Beam Search (MBS) algorithm, which is efficient for learning genome-phenome relationship using BNs. The MBS algorithm was developed to use the extended greedy search and learn DAG models that contain two or more predictors in the epistatic interaction [41]. The algorithm has been applied to GWAS data and successfully discovered the epistatic interaction of SNPs that have causal effect on Late Onset Alzheimer disease (LOAD) [41, 42]. MBS can be used to learn from data the interactive relationship among a subset of predictors that together can have a causal effect on a clinical feature. In many applications we need to investigate how subsets of a large dataset affect a target such as a clinical feature. For example, in the case of genome-wide association study (GWAS) datasets, we have data on perhaps millions of single nucleotide polymorphisms (SNPs) and diseases status. We want to determine subsets of SNPs that interact to affect the disease. It is intractable to look at all subsets even if we limit the subset size to 4 or 5. MBS ameliorates this problem by initiating a beam starting with every predictor (SNP). On that beam it does a greedy search by adding the predictor that increases the score of the model the most. When no predictor increases the score, it does greedy backward search deleting the predictor that increases the score of the model the most. Such a search is tractable even when the number of predictors is in the thousands. We know of no other method that uses this unique approach of doing multiple greedy searches.

However, the MBS algorithm had not been used to identify interacting predictors using genomic expression profile data, and this is the first application to genomic expression data. We hypothesize that the MBS algorithm will learn from data the joint probabilistic effect of miRNA and mRNA on cancer pathogenesis, and therefore successfully discover the interacting pairs of miRNA-mRNA. Individually, a given miRNA and mRNA might exhibit modest or no marginal statistical association with cancer pathogenesis. However, if they are interacting, taken together they would exhibit stronger statistical association, which means the MBS algorithm could discover the interacting pairs.

## Methods

First, we discuss the BN DAG model we used to represent a causal miRNA-mRNA interaction model. Then we present the algorithm we used to search over all possible models. Next we show the way we computed the posterior probability of each model. Finally, we describe how we collected miRNA-mRNA breast cancer-associated interactions, the datasets we used in the study, and how we pre-processed the data.

### The DAG model representing interaction

**Fig 2:**
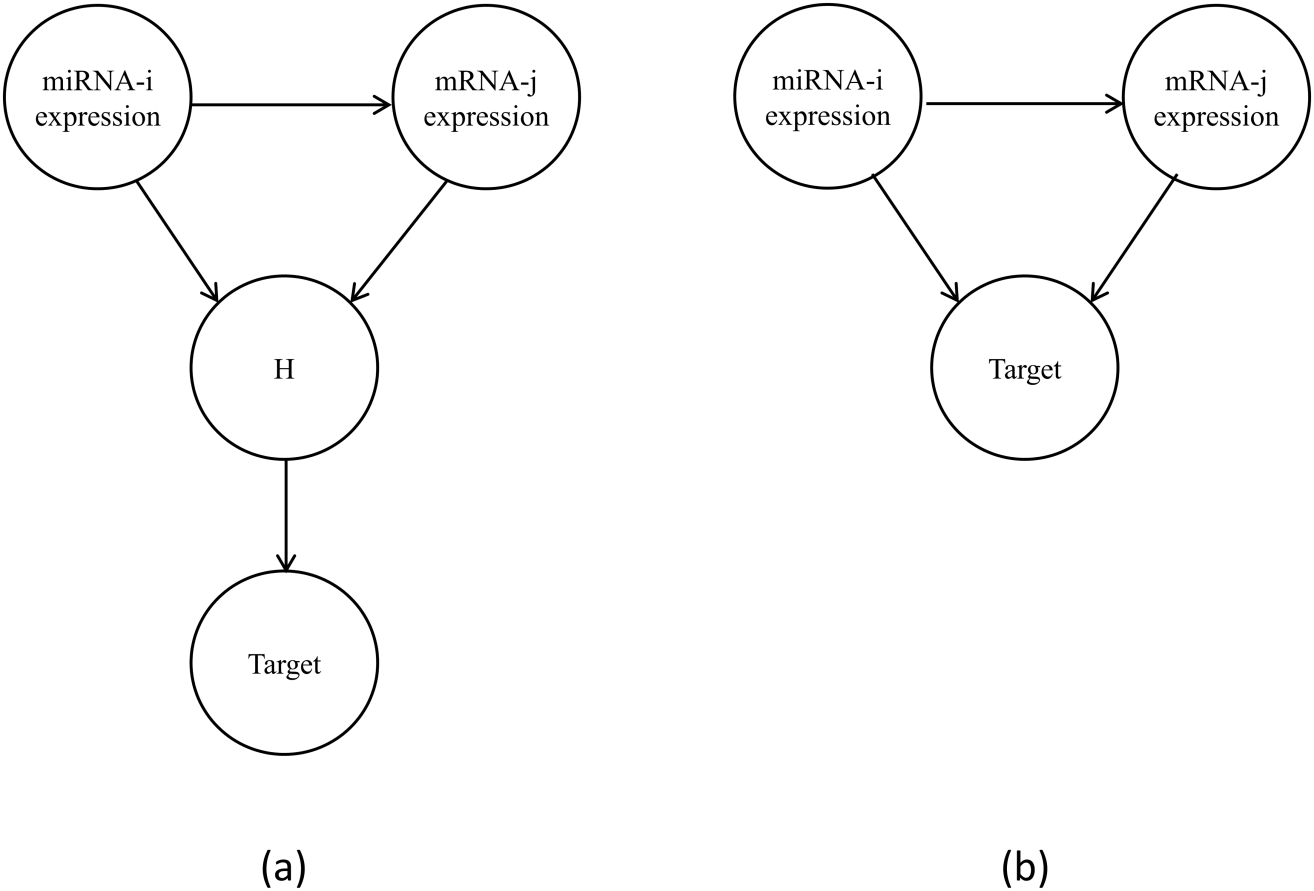
The model for the effect of miRNA-i on the target through the hidden variable. (a). The model including the hidden variable *H*, (b) The interaction model learned from data.

Measurements of miRNA and mRNA expression levels are at a snapshot in time, and therefore can miss some of the effect miRNA has on protein production including translational inhibition [43]. So, we model the causal effect of a particular miRNA on a target *T*, (e.g. cancer subtype), through its effect on a particular mRNA, using the DAG model in Fig 2(a). In that model, node miRNA-i represents our measured expression level of miRNA-i, and node mRNA-j represents our measured expression level of mRNA-j. There is an edge from miRNA-i to mRNA-j because the level of miRNA-i is assumed to affect the level of mRNA-j. However, as discussed above, the relationship among miRNA-i, mRNA-j, and the target is complex, and the miRNA-i expression level might have an influence on the effect that the mRNA-j expression level has on the target. This is similar to genetic epistasis, in which the effect of one gene is dependent on the presence of an allele of another gene. In the same way, we postulate that the effect of mRNA-j on the target is dependent on the expression of miRNA-i. We model this phenomenon using a hidden variable *H*, where the two measured expression levels interact to produce *H*, which exerts an effect on the target. Since we do not observe *H*, we learn the possible interaction by investigating the model in Fig 2(b). Note that in Fig 2(b) we do not show a possible edge from miRNA-I to mRNA-j because we are not investigating the existence of the edge. The overexpressed miRNAs in human tumors can affect the expression level of mRNAs. The measured expression levels of miRNAs and mRNAs interact with each other and exert an effect on cancer subtype.

### Identification of miRNA-mRNA interactions that cause cancer pathogenesis using MBS

The MBS algorithm begins with the empty DAG model and does greedy search starting with every miRNA and mRNA, rather than just a single miRNA or mRNA. From these initial predictors, every miRNA-miRNA, mRNA-mRNA, or miRNA-mRNA pair will be evaluated to investigate whether the pair increases the BDeu score of the DAG model. Many (but not all) interactions will be detected by extended greedy search in this step from a single miRNA or mRNA to every miRNA-mRNA, and the algorithm will create edges from the pair of miRNA-mRNA to cancer clinical feature. The algorithm will greedily add any miRNAs or mRNAs to the current predictors so far chosen while the addition increases the BDeu score, and create edges from the predictors to the target. Then, it will greedily delete any edge for miRNAs or mRNAs from the pair of miRNA-mRNA while the deletion increases the BDeu score. MBS reports the highest scoring models with any pairs of miRNA-miRNA, mRNA-mRNA, or miRNA-mRNA. But, we take only the pair of miRNA-mRNA, and ignored miRNA-miRNA or mRNA-mRNA because we are interested in only such pairs. MBS is a heuristic search algorithm which can search over the candidate DAGs with the large number of predictors. In its worst case, the time complexity is *O*(*n*^3^), where *n* is a total number of miRNAs and mRNAs.

We applied the MBS algorithm to the pre-processed miRNA and mRNA expression profile and clinical datasets. The MBS algorithm searches over possible models, scores each model using the BDeu score, and returns high-scoring models. We ran MBS with BDeu α = 54.

### Using Bayesian Network Posterior Probability (BNPP) to validate a DAG model

The BNPP method, developed by Jiang et.al [44], was used to compute posterior probability of DAG models that were already discovered or conjectured by the MBS algorithm. The BNPP can function as a validation to provide more confidence for the DAG models identified by a search algorithm when it is used with the BDeu scoring method together [45].

The posterior probability of a model *M* given Data is computed using Bayes’ Theorem as follows:

$$P(M|Data)=\frac{P(Data|M)P(M)}{P(Data)}$$ (2)

The *P*(*Data|M*) term is calculated using the BDeu score (Eq 1) with a particular choice of α. The *P*(*M*) term is the prior probability of *M*. The DAG model discovered by the MBS algorithm like Fig 2b includes the possibility of four competing models; miRNA and mRNA together are associated with target, two predictors with no marginal effect on target, either miRNA or mRNA by itself has an effect on target. The BNPP calculation considers these multiple competing models. The formula of the actual posterior probability of the models is as follows:

$$P(M_{ij}|Data)=\frac{P(Data|M_{ij})P(M_{ij})}{P(Data\left|M_{ij})P(M_{ij})+P(Data\right|M_{0})P(M_{0})+\Sigma_{k}P(Data|M_{K})P(M_{K})}$$ (3)

*M*_*ij*_, the model that miRNA and mRNA together have association with target

*M*_0_, the model that neither miRNA nor mRNA has association with target

*k*, sums over the two models that either miRNA or mRNA has association with target

We calculated the BNPP of miRNA-mRNA interaction models discovered by the MBS algorithm. We assigned non-informative prior probability 0.25 (1 divided by 4) to the four competing models. From the miRNA-mRNA interaction models with the BNPP, we selected the models that have the BNPP > 0.95 as true DAG models given the data.

### Breast cancer-associated miRNA-mRNA interactions

We collected known miRNA-mRNA breast cancer-associated interactions from miRNA-disease databases and a literature search. The databases we investigated are miRCancer (http://mircancer.ecu.edu/) [46], miR2Disease (http://www.mir2disease.org/) [47] and the Human microRNA Disease Database (HMDD, http://cmbi.bjmu.edu.cn:8080/html/tools/hmdd2.html) [48]. The review papers we referenced are studies done by O'Day et al. [49], Yu et al. [50], Zhang et al. [51], Luqmani et al. [52], and van Schooneveld et al. [53]. The databases and the review papers were manually curated to collect pairs of miRNA-mRNA interactions that are biologically verified by lab experiments only. We did not include the interactions predicted by computational algorithms. From the databases and papers, we collected information regarding the change of expression levels and the role of miRNAs in breast cancer progress, such as oncogenic, tumor suppressive functions, and promoting tumor invasion. The function and role of the breast cancer-associated mRNAs (cancer genes) were investigated using the Atlas of Genetics and Cytogenetics in Oncology and Haematology (http://atlasgeneticsoncology.org/) [54]. The information we could not find from the databases or review papers was obtained by additional literature search in PubMed. We thoroughly examined individual reference papers referred in the databases and the review papers to confirm the expression level and role of miRNAs and mRNAs in breast cancer. As a result of this work, we made the list of “breast cancer-associated miRNA-mRNA interactions” to use as standard information for the selection of the breast-cancer-associated miRNAs and mRNAs from publically available expression datasets.

### Expression profile data of miRNA and mRNA

We collected RNA-seq data of miRNAs and mRNAs expression for histologically defined breast cancer including normal tissues adjacent to the cancer from publicly available repositories, such as TCGA now available at Genomic Data Commons (https://portal.gdc.cancer.gov/repository/) and NCBI Gene Expression Omnibus (GEO, http://www.ncbi.nlm.nih.gov/geo/). We included data only from samples in which expression profile of miRNA and mRNA were measured on the same patient, and also collected clinical data features, such as tumor vs normal, ‘metastatic tumor indicator (yes or no), and molecular subtypes (e.g. luminal A, luminal B, triple negative/basal-like, and ERBB2/HER2+ types). The calculated expression signals in miRNAs RNA-seq data from TCGA were relative expression level (Level 3) that are already normalized to Reads Per Kilobase of transcript per Million mapped (RPKM) reads. The transcripts for all sequence reads were already mapped to a particular miRNA based on miRBase ver.20 (http://mirbase.org/) [55] and Human Genome ver. 19 (GRCh37). The transcripts for all sequence reads of mRNAs were aligned to NCBI gene symbols by MapSplice method [56] based on Human Genome ver. 19 (GRCh37) and quantified by RSEM method [57].

In contrast to TCGA data, datasets downloaded from GEO are all microarray data. We used the expression profile data that was provided in the ‘series_matrix.txt’ file, which contains the binary logarithm (log base 2) expression values normalized beforehand in each laboratory that provided the data to GEO. We mapped the probe set IDs to miRNA names or NCBI gene symbols based on microarray platform mapping data that is provided by GEO. We excluded from analysis some patients where either TCGA or GEO expression data were missing for molecular subtype data. The gene expression profile data and patient clinical data (de-identified) that are used in this research are all available in the public domain. Therefore, this research is exempt from any requirement for ethical review.

### Pre-processing of the expression profile data

**Fig 3:**
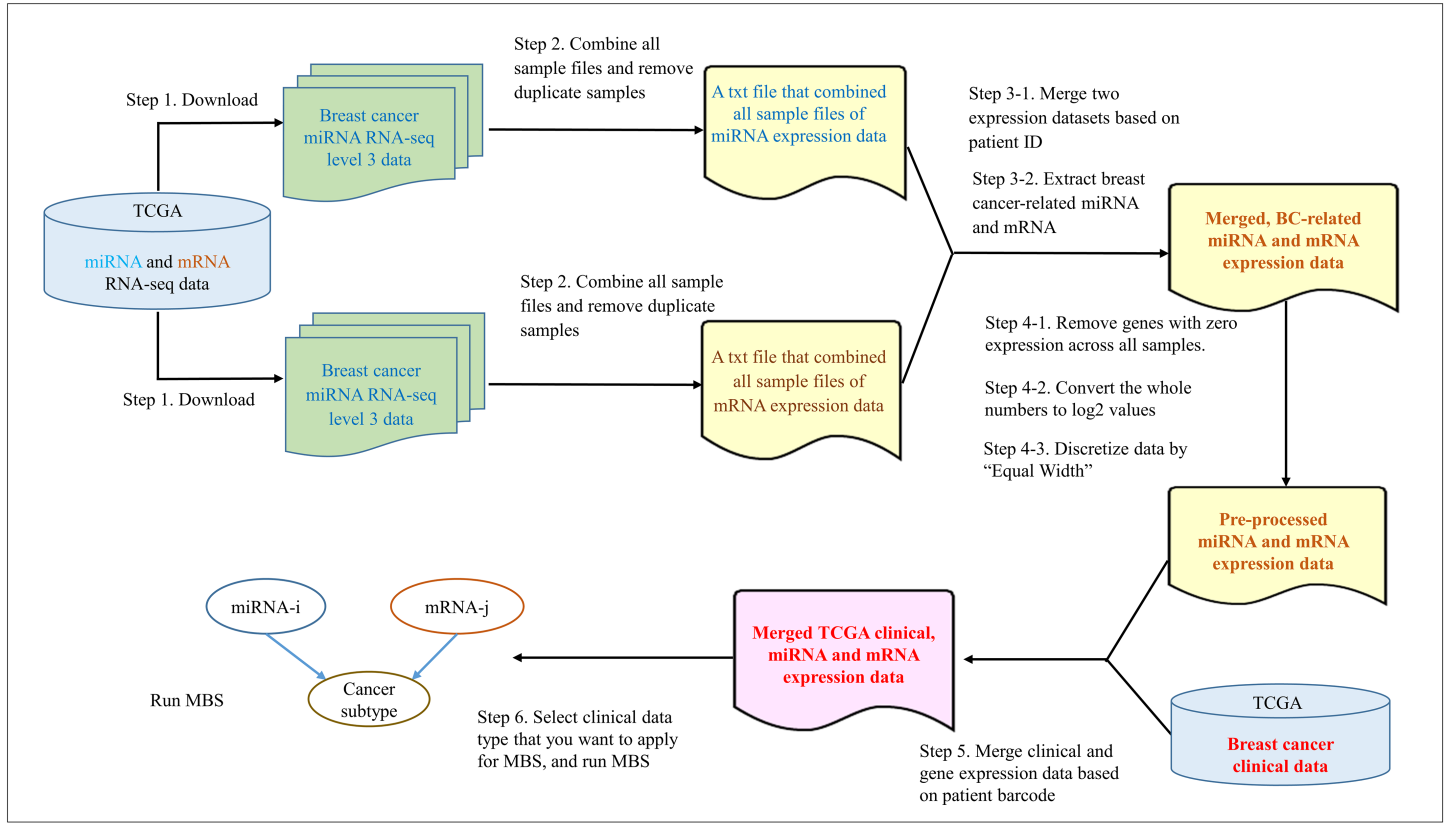
Schematic diagram for the pre-processing workflow and learning DAG models on microRNA and mRNA expression profile data.

From the collected expression profile data, we combined miRNA and mRNA datasets by common patient IDs, and extracted breast cancer-associated miRNAs and mRNAs from each dataset based on the list of interaction pairs we curated. We processed these data to construct discrete BNs. We removed miRNAs and mRNAs of which expression level is zero across all patients, and converted the relative expression values to the binary logarithm (log base 2) values using R code. Also, we discretized the continuous value of expression levels in each miRNA or mRNA to equal-width bins using the unsupervised discretization method available in the Information-Theoretic Measures (Infotheo) R package [58]. The processed expression dataset was merged with clinical data by using R code to indicate clinical features for each patient. The Infotheo discretization and binary logarithm conversion were performed using R (version 3.2.3) [59]. TCGA data required these complex pre-processing steps but GEO data did not. The complete pre-processing workflow for TCGA data is shown in Fig 3.

## Results

### Standard information of breast cancer-associated miRNA-mRNA interactions

We made a list of the breast cancer-associated miRNA-mRNA interactions based on the databases and review papers of miRNA-mRNA interactions in breast cancer. We annotated the expression level status (up- or down-regulated) and functions of miRNAs and mRNAs in breast cancer (S1A Table), and cited a reference paper for every interaction pair. The miRNAs up-regulated in breast cancer act as oncogenic miRNAs, and the down-regulated mRNAs as tumor suppressive miRNAs. The total number of miRNA-mRNA interaction pairs is 204 after removing the interaction pairs overlapping between databases and review papers. And, the numbers of unique miRNAs and mRNAs are 95 and 118, respectively. We used this list of miRNA-mRNA interactions as the standard information to extract breast cancer-associated miRNAs and mRNAs from expression profile datasets that we collected.

### Collection of expression profile data and pre-processing the data

We collected miRNA and mRNA expression profile data and patient’s clinical data in breast cancer from three datasets; one from TCGA, and 2 from NCBI GEO. Table 1 lists the dataset IDs, RNA-seq or microarray platform information, and the number of available patients after combining miRNA and mRNA expression data and merging with a cancer clinical features. We accomplished this pre-processing work for a total of 1,207 (1,131 tumor and 76 normal) breast cancer patients from the three datasets. The TCGA dataset has the largest number of patients; 747 tumor samples and 76 normal samples (a total of 823 patients). Out of 747 tumor samples, we counted 297 luminal A, 72 luminal B, 81 triple negative/basal-like, and 25 ERBB/HER2+ subtype patients, but 272 patients do not have molecular subtype data. The two datasets obtained from GEO are GSE58215 and GSE19783; out of 283 tumor samples in GSE58215 dataset, we counted 121 luminal A, 69 luminal B, 36 triple negative/basal-like, and 32 ERBB2/HER2+ subtype patients; in GSE19783 dataset out of 101 tumor samples, we counted 41 luminal A, 12 luminal B, 15 triple negative/basal-like, and 17 ERBB2/HER2+ subtype patients.

**Table 1:** Summary of microRNA and mRNA expression datasets in breast cancer. (Patient that have both miRNA and mRNA expression data.).

No.	Dataset ID	High-throughput experiment platform		No. of samples by cancer phenotype		No. of samples by 'metastatic_tumor_indicator'		No. of samples by breast cancer molecular subtype					No. of mRNAs overlapping with the list in breast cancer-associated miRNA and mRNA	
		miRNA expression data	mRNA expression data	Tumor	Normal	Yes	No	Luminal A	Luminal B	Triple negative (basal-like)	ERBB2/HER2+	Not available	miRNA	mRNA
1	TCGA	IlluminaHiSeq miRNASeq	IlluminaHiSeq RNASeqV2	747	76	12	363	297	72	81	25	272	103	113
2	GSE58215	Agilent-029297 Human miRNA Microarray v14 Rev.2	Agilent-028004 SurePrint G3 Human GE 8x60K Microarray	283	0			121	69	36	32	25	116	64
3	GSE19783	Agilent-019118 Human miRNA Microarray 2.0	Agilent-014850 Whole Human Genome Microarray 4x44K	101	0			41	12	15	17	16	131	68
	Total Counts			1131	76	12	363	459	153	132	74	313	47*	48*

Based on the standard information of breast cancer-associated miRNA-mRNA interactions, which has 96 of unique miRNAs and 118 of unique mRNAs (S1 Table), we extracted 103 out of 1,046 miRNAs, and 113 out of 12,671 mRNAs from TCGA expression profile data; from GSE58215 dataset, we extracted 116 out of 421 miRNAs, and 64 out of 14,305 mRNAs; from GSE19783 dataset, we extracted 131 out of 469 miRNAs, and 68 out of 17,175 mRNAs (Table 1). The reason the datasets have more than 96 miRNAs is because they contain many ‘star-strand’ miRNAs (e.g. hsa-miR-214* or hsa-miR-101*) and -5p / -3p miRNAs (e.g. miR-101-5p or miR-101-3p). Some precursor miRNAs give rise to two functional mature strands, and the less predominant miRNA is ‘star-strand’ miRNA. If there is no sufficient information to determine which strand is predominant or ‘star-strand’, the two strands of miRNAs are named as miR-101-5p (from the 5' arm) and miR-101-3p (from the 3' arm). Also, we included many miRNAs which fall into the same miRNA family to the pre-processed data. miRNA family is a group of miRNAs that encoded from the common ancestor and generally the members from the same family execute similar biological and physiological functions [60].

### miRNA-mRNA interactions associated with breast cancer phenotype

We ran our MBS algorithm on the TCGA data to discover the miRNA-mRNA interactions associated with breast cancer (tumor vs. normal). The MBS algorithm checked a total of 94,176 DAG models and discovered 104 models of miRNA-mRNA interactions with high BDeu score. But, just 28 out of the 104 interaction models had the BNPP > 0.95 (Table 2, top section). The most of the 28 models had high posterior probabilities (0.97~0.99), which means the interaction models have very high likelihood. However, their ranks of the BDeu score in the interaction model are not correlated with the ranks of the BNPP. In the 28 interaction models, hsa-miR-21 most frequently appeared to interact with various breast cancer genes (14 out of 28 interactions models), followed by hsa-miR-10b (8 interaction models), hsa-miR-96 (3 interaction models), hsa-miR-340 (1 interaction model), hsa-miR-365b (1 interaction model), and hsa-miR-448 (1 interaction model). We summarized the interaction models between those miRNAs and oncogenes (indicated in bold) or tumor suppressors (indicated in italic) in Table 2. We found that hsa-miR-21 and has-miR-96 forms the interaction model mostly with oncogenes, but hsa-miR-10b forms three interaction models with tumor suppressors, FOXO1, SERPINB5, and STARD10, to have statistical dependency on breast cancer.

**Table 2:** List of miRNA-mRNA interactions that are associated with breast cancer phenotype, metastatic tumor indicator, and molecular subtype.

No.	miRNA	mRNA	BDeu score (natural logarithm)	BNPP
miRNA-mRNA interactions that are associated with breast cancer (tumor vs. normal)				
1	hsa-miR-448	RYR3	-131.06	0.99
2	hsa-miR-21	ADIPOR1	-139.33	0.99
3	hsa-miR-96	**FN1**	-139.52	0.99
4	hsa-miR-21	PPM1F	-140.18	0.99
5	hsa-miR-21	**MERTK**	-145.41	0.99
6	hsa-miR-21	ELAVL1	-147.12	0.99
7	hsa-miR-21	*HOXA10*	-148.04	0.99
8	hsa-miR-21	PIK3R2	-148.87	0.99
9	hsa-miR-10b	**MET**	-151.41	0.99
10	hsa-miR-21	**TGFBR2**	-152.05	0.99
11	hsa-miR-10b	**WASF3**	-152.62	0.99
12	hsa-miR-10b	UBE2I	-152.64	0.99
13	hsa-miR-21	**ZEB2**	-152.72	0.99
14	hsa-miR-21	**CCND2**	-154.13	0.99
15	hsa-miR-21	TIMP3	-154.30	0.99
16	hsa-miR-21	HOXD4	-154.96	0.99
17	hsa-miR-21	**ESR1**	-155.02	0.99
18	hsa-miR-96	**C1QTNF6**	-155.04	0.99
19	hsa-miR-10b	SATB1	-155.15	0.99
20	hsa-miR-21	**NOTCH1**	-155.35	0.99
21	hsa-miR-10b	*FOXO1*	-155.99	0.99
22	hsa-miR-21	HOXD10	-157.45	0.97
23	hsa-miR-10b	*SERPINB5*	-160.61	0.99
24	hsa-miR-10b	CSNK2A2	-164.28	0.99
25	hsa-miR-365b	RYR3	-164.30	0.99
26	hsa-miR-340	RYR3	-167.24	0.98
27	hsa-miR-10b	*STARD10*	-167.44	0.99
28	hsa-miR-96	**TGFB1**	-171.57	0.99
miRNA-mRNA interactions that are associated with metastatic tumor indicator				
1	hsa-miR-448	MCL1	-61.56	0.99
2	hsa-miR-124-3	MCL1	-62.70	0.99
3	hsa-miR-448	SETDB1	-64.25	0.99
4	hsa-miR-448	*PTEN*	-65.08	0.97
5	hsa-miR-448	SNAI1	-65.20	0.97
miRNA-mRNA interactions that are associated with breast cancer molecular subtype				
1	hsa-miR-448	**ESR1**	-359.70	0.99
2	hsa-miR-661	**ESR1**	-360.01	0.99
3	hsa-miR-124-3	**ESR1**	-376.64	0.97

Note: The BDeu score and BNPP were rounded off to the second decimal place; The table is sorted by the descending order of the BDeu score in each section; Oncogenes are indicated in bold and tumor suppressors are indicated in italic.

### miRNA-mRNA interactions associated with tumor metastasis and molecular subtypes

We applied the MBS algorithm to the TCGA expression profile data with metastatic tumor indicator data. The MBS algorithm checked a total of 47,956 DAG models and discovered 114 models of miRNA-mRNA interactions with their BDeu scores. But, just 5 out of the 114 models had the BNPP > 0.95 (Table 2, middle section). The MBS result showed that hsa-miR-448 most frequently appeared to form interaction models with 4 different mRNAs, and hsa-miR-124-3 composed an interaction model with MCL1.

The MBS algorithm was applied to the TCGA expression profile data with breast cancer molecular subtype data; luminal A, luminal B, triple negative/basal-like, and ERBB2/HER2+. The MBS algorithm checked a total of 79,826 DAG models and discovered 85 models of miRNA-mRNA interactions with their BDeu scores. But, just 3 out of the 85 interaction models had the BNPP > 0.95 (Table 2, bottom section). The MBS output showed that ESR1, transcription factor and proto-oncogene frequently overexpressed in breast cancer, is the only mRNA that interacts with hsa-miR-448, hsa-miR-661, and hsa-miR-124-3 for the probabilistic dependency on breast cancer molecular subtype.

### Validation of miRNA-mRNA interactions discovered in TCGA datasets

GSE19783 and GSE58215 datasets have miRNA and mRNA expression profile data of breast cancer patients with molecular subtypes. We ran the MBS algorithm on these GEO datasets to validate the miRNA-mRNA interactions identified in TCGA dataset. The MBS algorithm checked a total of 123,575 interaction models and discovered 85 models in the GSE19783 dataset, and checked a total of 58,032 interaction models and discovered 114 models in the GSE58215 dataset, respectively. But, just 12 out of the 85 models in the GSE19783 result and 3 out of the 114 models in the GSE58215 result had the BNPP > 0.95 (S1B Table). The MBS results we got from the GSE58215 was similar to one from TCGA data; ESR1 and BIRC5 were the main mRNAs that form interaction models with miRNAs. On the other hand, the MBS result in the GSE19783 dataset was quite different from one in TCGA or GSE58215 dataset. IGF1R, BCL2, and MET, which promotes breast cancer progression and tumor metastasis, composed interaction models with various miRNAs. The reason the MBS outputs in the GEO datasets are quite different from the result in the TCGA dataset is probably because the GEO datasets have small number of breast cancer patients. In the MBS result of the GEO datasets, we did not get interaction models that include hsa-miR-124 and hsa-miR-448 because the GEO datasets do not originally contain the miRNAs in the expression profile data.

For all of the mRNAs that the MBS algorithm discovered as the interaction models in TCGA and GEO datasets, we categorized them based on KEGG pathway gene signatures. We identified that many mRNAs belonged to specific categories related to cancer biological process, for example, PATHWAT_IN_CANCER, CELL_CYCLE, APOPTOSIS, BREAST_CANCER, and TRANSCRIPTIONAL_MISREGULATION_IN_CANCER (Table 3). These results affirmed that hsa-miR-21, hsa-miR-96, hsa-miR-10b, hsa-miR-340, hsa-miR-365b, hsa-miR-124, and hsa-miR-448 play an important role by interacting with various breast cancer genes which are associated with tumor metastasis and molecular subtypes.

**Table 3:** The result of categorization on KEGG pathway gene signature for the mRNAs interacting with miRNAs associated with breast cancer pathogenesis.

Gene symbol	Gene description by Molecular Signatures Database (MSigDB)
KEGG: PATHWAT_IN_CANCER	
IGF1R	Insulin-like growth factor 1 receptor
BCL2	B-cell CLL/lymphoma 2
BIRC5	Baculoviral IAP repeat-containing 5 (survivin)
PTEN	Phosphatase and tensin homolog
FN1	Fibronectin 1
FOXO1	Forkhead box protein O1
PIK3R2	Phosphoinositide-3-kinase, regulatory subunit 2
TGFBR2	Transforming growth factor, beta receptor II (70/80kDa)
TGFB1	Transforming growth factor, beta 1 (Camurati-Engelmann disease)
MET	Met proto-oncogene (hepatocyte growth factor receptor)
KEGG: APOPTOSIS	
BCL2	B-cell CLL/lymphoma 2
PIK3R2	Phosphoinositide-3-kinase, regulatory subunit 2
KEGG: CELL_CYCLE	
TGFB1	Transforming growth factor, beta 1 (Camurati-Engelmann disease)
KEGG: TGFb_SIGNALLING	
TGFBR2	Transforming growth factor, beta receptor II (70/80kDa)
TGFB1	Transforming growth factor, beta 1 (Camurati-Engelmann disease)
KEGG: ERBB_SIGNALLING	
PIK3R2	Phosphoinositide-3-kinase, regulatory subunit 2
KEGG: BREAST_CANCER	
NOTCH1	Notch1
ESR1	Estrogen receptor 1
KEGG: TRANSCRIPTIONAL_MISREGULATION_IN_CANCER	
HOXA10	Homeobox A10

## Discussion

Indirectly and directly, miRNAs regulate many oncogenes, tumor suppressors, and transcription factors that mediate tumor progression, proliferation, and metastasis. The dysregulation of miRNA expression level is implicated to the alteration of target mRNA expression level, and in the long run it causes the phenotypic abnormalities in human cancer. Multiple miRNAs collectively regulate one or more pathways in gene regulatory networks [61]. The collective relationship between multiple miRNAs and multiple mRNAs will be constructed to possess similar biological functions and effects on cancer pathogenesis. Correct interpretation of the relationship between miRNAs and mRNAs provides more useful clinical insights into gene regulation rather than individual interpretation of miRNA and mRNA. Therefore, identification of such miRNA-mRNA interactions that have biological consequences on cancer pathogenesis requires integrative analysis of miRNA-mRNA expression profile data from cancer patients. In addition, the statistical interaction of miRNA-mRNA in gene regulatory network cannot be predicted by a simple statistical test, such as chi-square test, that evaluates the effect of individual predictors on the target variable.

Several bioinformatics methods and algorithms have been proposed to analyze miRNA-mRNA expression profile data and identify the interactions that have regulatory functions and biological effects on tumor development [34, 62–65]. Fu et al.[66] used computational and bioinformatics approach. They combined microarray data of miRNA and mRNA, and used Pearson’s correlation analysis to find significant miRNA-mRNA interactions. From the significant interaction pairs, they identified colorectal cancer specific miRNA-mRNA regulatory interactions based on the knowledge in miRNA-mRNA prediction database, TargetScan [21] and miRanda [67].

Just a few researchers used Bayesian approach and expression profile data to identify miRNA-mRNA interactions and mRNAs. Liu et al. [33] integrated expression profiles of miRNAs and mRNAs according to phenotypic conditions and then built BNs on the separate data sets. Interactions of miRNA-mRNA were identified on individual data sets and integrated by BN averaging procedure. Stingo et al. [64] integrated expression data of miRNAs and potential target mRNAs, and used a Bayesian graphical modeling to construct the miRNA regulatory network. This Bayesian approach has advantage in integrating multiple data sources and inferring network. Huang et al. [68] integrated miRNA and mRNA expression profile data across 88 tissues and cell types, and used GenMiR++, which is a Bayesian inference algorithm, to identify the interaction network between 1,597 mRNAs and 104 human miRNAs. This study demonstrated that application of Bayesian statistics algorithm to the simultaneous profiling data of miRNA and mRNA expression is an effective strategy to identify functional miRNAs-mRNA interactions, although the molecular hybridization depending on sequence complimentary between miRNAs and mRNAs is essential to exert its biological functions. This study did not associate miRNA and mRNA genomic data with cancer pathogenesis for the identification of miRNA-mRNA interactions, but they associated cancer pathogenesis in the validation for the interaction pairs discovered by the GenMiR++ algorithm. They experimentally demonstrated that let-7b was down-regulated in retinoblastoma and the target mRNAs of let-7b, such as CDC25A and BCL7A, were over-expressed.

Many methods including GenMiR++ depend on prior knowledge, such as lab confirmed interactions, to associate the identified miRNA-mRNA interactions with cancer phenotype, while MBS uniquely learns collectively relationship between group of miRNAs and group of mRNAs that together have strong causal effects on cancer clinical features. Compared to other studies, the major advantages of our study are that we modeled causal relationship between miRNA-mRNA expression and cancer clinical data using BN, and identified miRNA-mRNA interactions that cause phenotypic abnormality in breast cancer patients using MBS. In addition, we calculated BNPP to validate the true interaction models discovered by the MBS algorithm. Learning genome-phenome relationship in BNs has the advantages to discover statistical interactions of genes from expression data, and examine causal relationship between genes and clinical features [69, 70]. Our hypothesis is that modeling miRNA-mRNA interactions using BNs and a good search algorithm will be required for finding the interactions that have stronger marginal effect on cancer clinical features when miRNAs and mRNAs are together than they exist individually. In this study, we integrated miRNA and mRNA expression profile data with human cancer clinical data, and applied the MBS algorithm to the BN models for learning the genome-phenome relationship.

According to the standard information we curated, many studies showed that dysregulation of miRNA expression level, especially hsa-miR-21, hsa-miR-10b, hsa-miR-96, is associated with tumor invasion, metastasis, migration and epithelial—mesenchymal transition (EMT) or tumor suppression through interaction with breast cancer genes [2, 22, 71–75]. Also, hsa-miR-340 is known to inhibit breast cancer cell migration and invasion [76], and suppression of hsa-miR-448 is known to be correlated with EMT induction in breast cancer [77]. The MBS algorithm discovered the oncogenes, such as ESR1, NOTCH1, TGFBR2, and TGFB1, the transcription factor HOXA10, and tumor suppressor PTEN to form statistical interaction with these miRNAs (Table 2, top section). Theses oncogenes, and the transcription factor are a bit studied that they interact with various miRNAs to promote breast cancer cell proliferation or tumor metastasis. TGFBR2 and TGFB1 are considered putative targets of miR-21 that cause metastasis and patient poor prognosis in breast cancer [2]. The overexpression of ESR1 and NOTCH1 is correlated with breast cancer progression and prognosis [78, 79]. A sequence variant on ESR1 affects miRNA-binding affinity for miR-453 and the interaction is significantly associated with breast cancer risk [80]. The expressions of NOTCH1 and miR-21 were positively correlated with colorectal cancer development [81]. HOXA10 is a transcription factor and has metastasis suppressive function in breast cancer.

**Fig 4:**
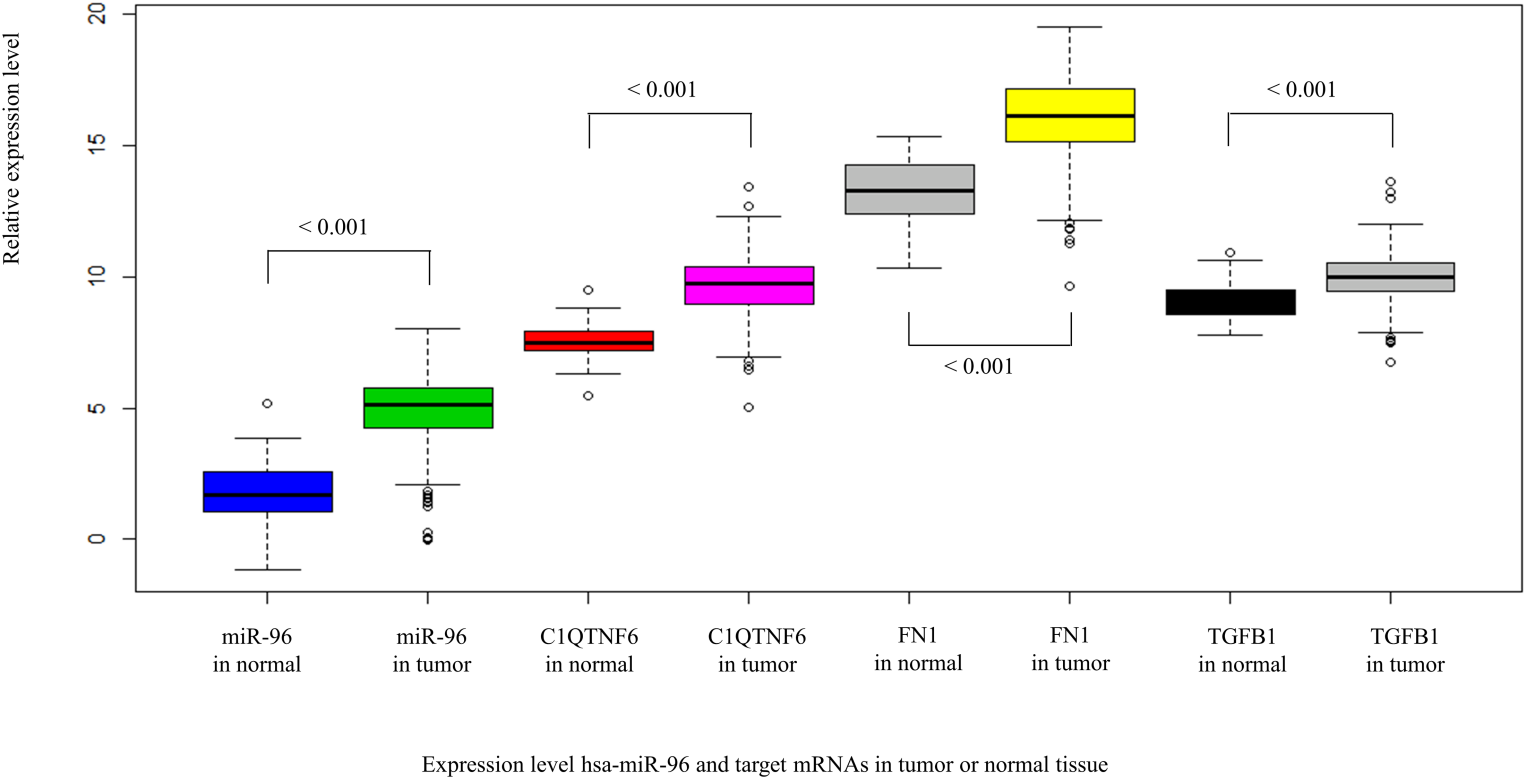
The expression level of hsa-miR-96 and its target mRNAs that are associated with breast cancer. The expression levels of miRNAs and mRNAs were represented in 76 normal tissue samples and 747 tumor patients. t-test p-values are indicated above the box plots.

The MBS algorithm discovered FOXO1, SATB1, SERPINB5, and STARD10 to form interaction models with hsa-miR-10b (Table 2, top section). FOXO1, SERPINB5, and STARD10 are putative breast cancer suppressor. FOXO1 is known to be regulated by miR-27a, miR-96, and miR-182 in breast cancer cells [82], SERPINB5 is regulated by miR-21 [83], and STARD10 is regulated by miR-661 [84]. SATB1 is known to interact with miR-448, and the suppression of miR-448 increased SATB1 expression and induced EMT of breast cancer cells [77]. Therefore, the interaction model between miR-10b and those genes can be new putative ones that are associated with breast cancer progression. Further analyses are required to elucidate the mechanism of the interaction between the miRNAs and oncogenes or transcription factors. In addition, three oncogenic mRNAs, FN1, C1QTNF6, and TGFB1, were predicted to form statistical interaction with hsa-miR-96 (Table 2, top section). It is known that hsa-miR-96 is overexpressed in breast cancer and promotes tumor proliferation and invasion by suppressing transcription factors FOXO1 and FOXO3a [75, 82]. FNI is known to induce tumor metastasis, and TGFB1 acts as proto-oncogene which can induce cell growth when mutated. If hsa-miR-96 directly interacts with C1QTNF6, FN1, and TFGB1 in breast cancer, the expression level of mRNAs should be down-regulated because the overexpressed hsa-miR-96 will suppress the mRNA’s expression. So, we further investigated the overall expression level of hsa-miR-96 and its target mRNAs between normal and tumor patients. Indeed, hsa-miR-96 was up-regulated in tumor patients, but all of interacting mRNAs were also up-regulated in tumor patients (Box plot and t-test p-value in Fig 4). This means that there is possibility that the interaction of hsa-miR-96 and the oncogenic mRNAs mediated by other genes, or the up-regulated oncogenic mRNAs affected hsa-miR-96 to be over-expressed, and the overexpressed hsa-miR-96 suppress FOXO1 and FOXO3a to promote tumor proliferation and invasion.

The MBS algorithm discovered the putative interactions between hsa-miR-448/hsa-miR-124-3 and various oncogenes that have causal effect on tumor metastasis in the TCGA expression profile data (Table 2, middle section). These miRNAs, hsa-miR-448 and hsa-miR-124-3, as well as hsa-miR-661, were predicted again by the MBS in TCGA data with breast cancer molecular subtypes (Table 2, bottom section) and composed interaction models with ESR1. Suppression of hsa-miR-448 and hsa-miR-124 (gene family name of miR-124-3) are known to induce EMT transition in breast cancer [77, 85]. hsa-miR-661 is known to be expressed at early time points of SNAI1-induced EMT and promote invasion of breast cancer cells by degrading two target genes, NECTIN1 (cell-cell adhesion protein), and STARD10 (lipid transferase) [84]. But, generally these miRNAs and their interaction with tumor gene or ESR1 are not studied enough.

All things considered, our results showed that the MBS algorithm is very efficient to learn genome-phenome data in BNs and the strength of our MBS algorithm that can discover miRNA-mRNA interactions that have stronger probabilistic dependency together associated with clinical feature. Our study has several limitations that we could not explain clearly. First, the miRNA-mRNA interactions in TCGA data identified in this study was validated by BNPP calculation, application of the MBS algorithm to GEO datasets, and literature support. It requires further experimental validation for the interaction models, which are identified by the MBS algorithm but not listed in the standard information, to examine their real interactions in biology. Second, the miRNA-mRNA interactions discovered by the MBS algorithm depended on only the expression level alteration of miRNAs and mRNAs associated with breast cancer clinical features. This can be one of the reasons why the MBS algorithm could not discover more miRNA-mRNA interaction pairs that are already listed in the standard information. Third, conversely, the MBS algorithm discovered many of new miRNA-mRNA interactions which are not listed in the standard information. This may be because the standard information is not complete to contain all available miRNA-mRNA interaction pairs. HMDD database needs to update to include more data form recent publications. There will be more miRNA-mRNA interaction pairs which are not included in review papers yet.

Our next step is applying the MBS algorithm to other cancer miRNA-mRNA expression profile data, such as prostate cancer and lung cancer, to see if the algorithm will be able to learn genome-phenome relationship in other cancers. In addition, we are going to test the MBS algorithm to identify interactions between different types of genomic data, for example, copy number variation (CNV) vs DNA methylation. If the two different types of genomic data have a larger marginal effect on cancer pathogenesis than each genomic data exists by itself, the MBS algorithm will discover the interesting new interactions.

Our results provide good candidates of miRNA-mRNA interactions to other breast cancer researchers who are interested in finding new interaction pairs that are biologically and clinically meaningful. The validation of the newly discovered interactions will inspire Cancer Biologists or Oncologists to investigate their clinical meanings.

## Supporting information

S1 Tables Data underlying this study. Table A. List of Breast cancer-associated microRNA-mRNA interactions. Table B. List of miRNA-mRNA interactions that are associated with breast cancer subtypes (NCBI GEO data).
(XLSX)
